# Epidemiological, Clinical, and Immunological Features of Ghanaian People-Living-with-HIV (Human Immunodeficiency Virus) and Molecular Proof of *Cystoisospora belli* in Their Stool Samples

**DOI:** 10.3390/pathogens14030212

**Published:** 2025-02-21

**Authors:** Hagen Frickmann, Fred Stephen Sarfo, Betty Roberta Norman, Martin Kofi Agyei, Albert Dompreh, Shadrack Osei Asibey, Richard Boateng, Edmund Osei Kuffour, Martin Blohm, Veronica Di Cristanziano, Torsten Feldt, Kirsten Alexandra Eberhardt

**Affiliations:** 1 Department of Microbiology and Hospital Hygiene, Bundeswehr Hospital Hamburg, 22049 Hamburg, Germany; 2 Institute for Medical Microbiology, Virology and Hygiene, University Medicine Rostock, 18057 Rostock, Germany; 3 Department of Medicine, Komfo Anokye Teaching Hospital, Kumasi 00233, Ghana; stephensarfo78@gmail.com (F.S.S.); branorman@yahoo.com (B.R.N.); martinagyei@yahoo.co.uk (M.K.A.); shakosbey19@gmail.com (S.O.A.); 4 Kwame Nkrumah University of Science and Technology, Kumasi 00233, Ghana; 5 Department of Clinical Microbiology, Komfo Anokye Teaching Hospital, Kumasi 00233, Ghana; adompreh@gmail.com (A.D.); richardboateng166@gmail.com (R.B.); 6 Laboratory of Retrovirology, The Rockefeller University, New York, NY 10065, USA; eosei@rockefeller.edu; 7 Department of Laboratory Medicine, Bundeswehr Hospital Berlin, 10115 Berlin, Germany; martin2blohm@bundeswehr.org; 8 Institute of Virology, Faculty of Medicine and University Hospital Cologne, University of Cologne, 50937 Cologne, Germany; veronica.di-cristanziano@uk-koeln.de; 9 Clinic of Gastroenterology, Hepatology and Infectious Diseases, University Hospital Düsseldorf, 40225 Düsseldorf, Germany; torsten.feldt@med.uni-duesseldorf.de (T.F.); k.eberhardt@bnitm.de (K.A.E.); 10 Department of Tropical Medicine, Bernhard Nocht Institute for Tropical Medicine & I. Department of Medicine, University Medical Center Hamburg-Eppendorf, 20359 Hamburg, Germany

**Keywords:** *Cystoisospora belli*, HIV, Ghana, immunology, epidemiology, clinical disease

## Abstract

*Cystoisospora belli* is a coccidian parasite commonly associated with enteric infections in immunocompromised individuals. The study was conducted to assess epidemiological, clinical, and immunological features of Ghanaian people living with HIV (human immunodeficiency virus) with and without antiretroviral therapy and molecular proof of *C. belli*-specific nucleic acid sequences in their stool samples. While *C. belli* was detected in 4.2% (*n* = 25) of the assessed HIV-positive patients, this was the case for only 1 (1.2%) Ghanaian control individuum without known HIV infection. Associations of cystoisosporiasis in Ghanaian HIV patients with reduced CD4+ T-lymphocyte counts and increased HIV viral loads, immune-activation as indicated by reduced CD4+/CD8+ T-lymphocyte ratios as well as higher expression of HLA-DR+ CD38+ on CD4+ T-lymphocytes, a symptom complex comprising diarrhea, weight loss and a reduced BMI, a trend towards not being on antiretroviral medication, and lacking access to food safety procedures like storing food in refrigerators were shown. The odds ratios (95% confidence intervals) of the associations were 4.47 (1.52–12.09) for the abundance of *C. belli* DNA and clinical diarrhea, 3.51 (1.42–9.12) for the abundance of *C. belli* DNA and CD4+ T-lymphocyte counts <200 cells/µL, and 3.66 (1.52–9.01) for the abundance of *C. belli* DNA and not having a refrigerator in the household. In conclusion, the assessment contributed to existing insight into the epidemiology of cystoisosporiasis in immunosuppressed individuals in resource-limited tropical high-endemicity areas. Chronic diarrhea among people living with HIV should prompt a diagnostic assessment for confirmation or exclusion of *C. belli* infections in such settings.

## 1. Introduction

Human *Cystoisospora* (formerly named *Isospora*) *belli* infections have been traditionally associated with co-occurring human immunodeficiency virus (HIV) infections or other kinds of severe immunosuppression [1]. In people living with HIV (PLWH), a pooled prevalence of 2.5% of *C. belli* infections has been globally estimated, with higher prevalence in low-income countries and in individuals with clinically apparent diarrhea [2]. Cystoisosporiasis has been globally reported in PLWH, however, with a quantitative dominance in tropical resource-limited settings and a particular focus in sub-Saharan Africa [2]. In international travel medicine, in contrast, *C. belli* infections are very rare [3]. Diarrhea is the common clinically presenting symptom in immunosuppressed individuals associated with this obligatorily intracellular parasite [4,5,6,7,8,9,10,11,12,13,14,15]. Impaired host T-lymphocyte response facilitates the invasion by intestinal parasites like *C. belli* [16]. A decreased CD4+ T-lymphocyte count <200 cells/µL was proposed as a risk factor for cystoisosporiasis [17,18], irrespective of co-occurring antiretroviral therapy [19], as well as for diarrhea and the abundance of enteric opportunistic pathogens in HIV-positive patients in general [20,21,22]. In AIDS (acquired immunodeficiency syndrome) patients with enteropathic disease, *C. belli* prevalence of up to 60% has been reported [23]. Further, coccidian parasites, including *C. belli* and microsporidia, account for about 50% of persistent diarrhea in immunosuppressed individuals [24,25], and chronic cystoisosporiasis is common in PLWH [26,27]. French authors described a 79% risk reduction for cystoisosporiasis in antiretrovirally treated PLWH compared to HIV-positive individuals not receiving therapy [28]. In the case of very low T-lymphocyte counts <50 cells/µL, however, this difference was not detectable anymore [28]. In the case of successful antiretroviral therapy and associated immune recovery, the risk of acquiring cystoisosporiasis can be considered low [28,29,30]. Of note, acalculous cholecystitis, as well as diffuse gall bladder infections and cholangiopathy, have been described as infrequently observed complications of cystoisosporiasis in immunosuppressed individuals [31,32]. Next to HIV infection, other causes of cellular immune alteration like HTLV-1 (human T-cell leukemia virus-1) infections or hemopoietic malignancy have been associated with fulminant and even therapeutically refractory cystoisosporiasis as well [33,34,35].

Cotrimoxazole is therapeutically indicated for the treatment of cystoisosporiasis [36] with the option of effective maintenance therapy in order to prevent relapse events in case of persisting immunosuppression [37]. Adequate immune reconstitution seems to be critical for treatment success, given that therapeutic failure has been reported in cases of PWLH with cytoisosporiasis and persisting low to moderate CD4 T-lymphocyte counts [38]. Ciprofloxacin was shown to be less effective for treating cystoisosporiasis and for its secondary prophylaxis; however, it still shows a therapeutic effect and may thus be considered for individuals who do not tolerate cotrimoxazole application [39]. Cases of successful therapy and secondary prevention of cystoisosporiasis with pyrimethamine with and without sulfadiazine have been described as well [40,41].

Traditionally, *C. belli*-associated enteritis was microscopically diagnosed [42]. In particular, small bowel biopsies were taken for this purpose in case of HIV-associated enteropathy [43]. In the case of cystoisosporiasis-induced cholecystitis, developmental stages of the coccidian parasite can be seen in gallbladder tissue sections [31]. While the width and length of *C. belli* oocysts vary, the length-width rate stays in the >1.2 range [44]. In contrast to most intestinal protozoan parasites, eosinophilia is strongly associated with *C. belli* infections [45]. High degrees of sequence conservation were reported for *C. belli*’s small subunit ribosomal RNA (rRNA), 5.8S rRNA, internal transcribed spacer 1 (ITS-1), and ITS-2 [44]. Consequently, the high diagnostic accuracy of ribosomal sequence-based real-time PCR for *C. belli* has recently been confirmed [46].

Patients from the tropics in general [15] and from sub-Saharan Africa in particular were shown to be at increased risk of being infected with *C. belli* [29], especially in case of late diagnosis of an HIV infection. Epidemiological information from Ghana, a West African country, exists for a population of pregnant women without specific preselection for immunosuppression. A prevalence of 0.3% was recorded [47]. A recent investigation with stool samples from the subpopulation of Ghanaian HIV patients by our group indicated an about 10x higher prevalence [46] compared to the general population [47]. *C. belli* oocysts have further been found on fresh vegetables as likely sources of infection [48].

The overall aim of this study was an evaluation of epidemiological, clinical, and immunological features of Ghanaian HIV patients with molecular evidence of *C. belli* in their stool samples. The assessed population comprised PLWH with and without antiretroviral therapy, thus allowing the influence of antiretroviral therapy on *C. belli* prevalence to be addressed.

## 2. Materials and Methods

### 2.1. Study Population

HIV-positive patients attending the HIV outpatient department of the Komfo Anokye Teaching Hospital (Kumasi, Ghana) were assessed as part of a study investigating associations of gastrointestinal and other pathogens with immunological and socio-demographic parameters in HIV positive and negative adults in Ghana [49]. About half of the included PLWH were on antiretroviral therapy during the study period, allowing for the assessment of the influence of treatment as well. An HIV-negative control population was investigated during the same time period of 12 months. All participants provided written informed consent prior to study participation. Demographic, socioeconomic, and clinical data were provided by filling in standardized questionnaires with the help of trained investigators.

### 2.2. Diagnostic Methods

Venous blood samples were taken to analyze the CD4+ T lymphocyte count locally using a FACSCalibur flow cytometer (Becton Dickinson, Mountain View, CA, USA). HIV-1 viral load was quantified by applying the Real-Time HIV-1 PCR system (Abbott Diagnostics, Wiesbaden, Germany).

By centrifugation of heparinized venous blood on a Ficoll/Hypaque (Biocoll Separating Solution, Biochrom AG, Berlin, Germany) density gradient, peripheral blood mononuclear cells (PBMCs) were collected. Washing of the cells was performed with phosphate-buffered saline. Subsequently, they were resuspended in Roswell Park Memorial Institute 1640 medium (Gibco Invitrogen, Carlsbad, CA, USA) supplemented with heat-inactivated fetal calf serum (Biochrom AG, Berlin, Germany). After cryopreservation, PBMCs were shipped to Germany using liquid nitrogen. Cell surface markers of immune activation were stained, as reported in detail elsewhere [50]. Flow cytometric data were measured with the help of an LSRII flow cytometer (BD Biosciences, Heidelberg, Germany), and the obtained data were analyzed by applying the software FlowJo (version 9.6.2, Tree Star, San Carlos, CA, USA).

Prior to nucleic acid purification, native stool sample aliquots were stored deep frozen at −80 °C. Nucleic acids were extracted with the QIAamp stool DNA mini kit (Qiagen, Hilden, Germany) as described by the manufacturer. Nucleic acid eluates were subsequently stored at −80 °C before the real-time PCR assessments were performed. The chosen laboratory-developed real-time PCR assay for cystoisosporiasis targeted a 90-base pair sequence of the ITS-2 sequence of *C. belli* [46,51]. Regarding the assay’s diagnostic accuracy, sensitivity of 100% and specificity of 99.8% had been estimated with a limit of detection of <10 copies per µL eluate as detailed elsewhere [46]. The in-house test was run on magnetic induction cyclers (MIC, Bio Molecular Systems Ltd., London, UK) applying 20 µL reaction volumes, including 5 µL eluate each. The used oligonucleotides comprised the forward primer Cys Ib-40-F (5′-ATATTCCCTGCAGCATGTCTGTTT-3′), the reverse primer Cys Ib-129-R (5′-CCACACGCGTATTCCAGAGA-3′) and the hybridization probe Cys Ib-81-P (5′-CAAGTTCTGCTCACGCGCTTCTGG-3′). The PCR reaction mix contained the HotStarTaq Mastermix (Qiagen, Hilden, Germany) with a final Mg^2+^ concentration of 5 mM. The concentrations of the oligonucleotides in the reaction mix were 60 nM for each primer and 200 nM for the probe. A PCR grade water-based negative control and a positive control consisting of a plasmid containing the *C. belli* sequence 5′-GGCGCTGTGGGGATATTCCCTGCAGCATGTCTGTTTCAGTGTCTCTGAAGTTTCAAGTTCTGCTCACGCGCTTCTGGGGGTGTCTCTGGAATACGCGTGTGGCAGTGTGACTGGATGTCTTGGGTGTTGAGAAACAAGCTACTTGTGCTTCTAGAAAGCCGAACGTCATCCGAAATAGTCACAGCGGCGTTTACGCGATCAAACAGTGTTGAGTTGTGTCCCGAACATCTTTG-3′ (NCBI GenBank accession number AF443614.1) inserted in a pEX-A128 vector backbone were used in each run. The real-time PCR run profile included an initial denaturation at 95 °C for 15 min followed by 45 cycles of denaturation at 95 °C for 15 s and annealing as well as amplification at 59 °C for 60 s. Subsequently, cooling down to 40 °C for 20 s was added. Control of sample inhibition was ensured using a Phocid herpes virus (PhHV) DNA-specific real-time PCR as reported elsewhere [52].

### 2.3. Statistics

Statistical analyses were conducted using the software R (version 4.4.2, R Foundation for Statistical Computing, Vienna, Austria). The comparison of categorical variables was performed using either the χ^2^ test or the Fisher exact test, as appropriate for the particular setting. Continuous variables were presented as median values (interquartile range, IQR) or mean values ± standard deviation (SD). They were compared by applying either the Wilcoxon rank sum test or the Student’s unpaired t-test. Multiple logistic regression analysis was performed using the R package ‘forestmodel’. The calculation of the Spearman rank correlation coefficient ρ was conducted in order to evaluate the relation between continuous variables. Two-sided *p*-values were provided. Statistical significance was accepted at α = 5%.

## 3. Results

### 3.1. Prevalence of C. belli Within the Stool Samples of the Study Population

**Figure 1:**
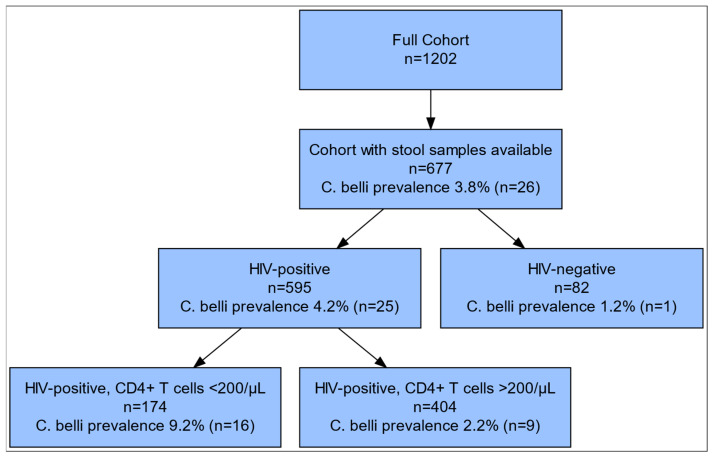
Flow diagram characterizing the study population and prevalence of *C. belli*.

A total of 1095 HIV-positive and 107 HIV-negative individuals were included in the original study. Residual stool samples for *C. belli* testing were available for 595 HIV-positive and 82 HIV-negative participants (Figure 1). The overall prevalence of *C. belli* was 3.8% (n = 26) in the entire study population, while it was 4.2% (n = 25) in HIV-positive patients and 1.2% (n = 1) in HIV-negative participants. Among HIV-positive patients, the detection rate was significantly higher in patients with CD4+ T lymphocyte counts below 200/μL compared to those with higher counts (9.2% [n = 16/174] vs. 2.2% [n = 9/404], *p* < 0.001).

### 3.2. Comparison of Demographic, Socioeconomic, and Clinical Characteristics of the HIV Cohort According to the Presence or Absence of C. belli in Their Stool Samples

No differences with regard to demographic factors, such as sex or age, were found in HIV-positive participants with or without colonization of *C. belli* (Table 1). Individuals carrying *C. belli* had significantly less frequent access to a refrigerator for food storage in their households than those without detection of this pathogen (48.0% vs. 73.9%, *p* = 0.009). Participants with *C. belli* had a lower body mass index (BMI) (21.3 vs. 23.2, *p* = 0.038) and were less often treated with cART than *C. belli* negative participants, although not reaching statistical significance (24.0 vs. 42.4, *p* = 0.095). Of note, no differences regarding the prophylaxis with cotrimoxazole were observed between the groups.

**Figure A1:**
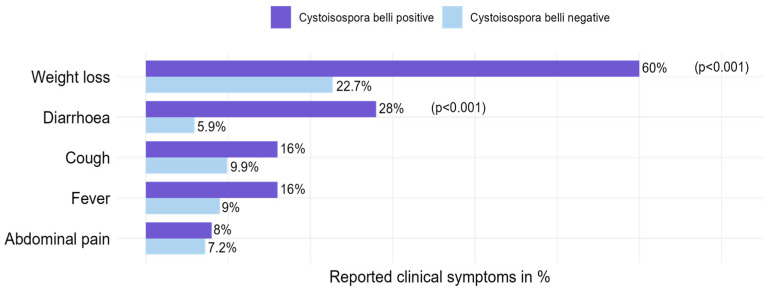
Prevalence of clinical symptoms during the last six months according to *C. belli* status among HIV-positive participants.

HIV-positive participants co-infected with *C. belli* reported the presence of several clinical symptoms significantly more often than those participants without detected *C. belli* (Appendix A Figure A1). In detail, 60.0% of co-infected participants described the presence of weight loss during the last six months (vs. 22.7% in HIV-positive *C. belli*-negative participants, *p* < 0.001), and 28.0% suffered from diarrhea (vs. 5.9%, *p* < 0.001). In contrast to this, other symptoms, such as acute fever, cough, or abdominal pain, occurred at a similar frequency in both groups.

### 3.3. Comparison of Virological and Immunological Characteristics of cART Naïve HIV Positive Participants Depending on the Abundance or Absence of C. belli in Their Stool Samples

Among HIV-positive cART naïve patients, 5.6% (n = 19/339) were found to be colonized with *C. belli*. Co-infected patients in this group had a significantly higher HIV-1 median viral load in log10 copies/mL (5.6 [5.2–6.1 IQR] vs. 4.1 [1.6–5.3 IQR], *p* < 0.001, Table 2) and a correspondingly lower CD4+ T cell count/μL (118 [77–359 IQR] vs. 348 [163–570 IQR], *p* = 0.001). The CD4+/CD8+ T cell ratio, which is inversely associated with immune activation in HIV, was significantly lower in *C. belli* carriers (0.2 [0.1–0.3 IQR] vs. 0.4 [0.2–0.7 IQR], *p* = 0.002). HIV-positive individuals with *C. belli* carriage had a significantly higher expression of HLA-DR+ CD38+ on CD4+ T lymphocytes as additional markers of immune activation (35.8 [28.9–38.7 IQR] vs. 17.5 [10.0–31.4 IQR], *p* = 0.001). No differences were found regarding the expression of markers of immune exhaustion, cell proliferation, and terminal differentiation or regarding markers of immune activation on CD8+ T lymphocytes when comparing cART naïve HIV patients with and without *C. belli* (Table 2).

### 3.4. Factors Associated with C. belli Co-Infection in the HIV-Positive Cohort

**Figure 2:**
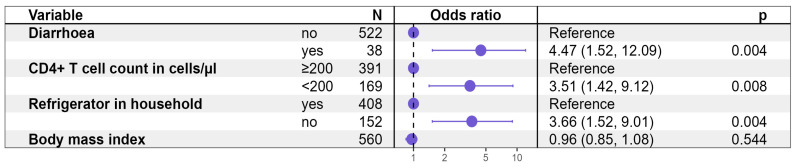
Logistic regression model: Factors associated with the detection of *C. belli* in fecal samples in HIV-positive participants.

Figure 1 demonstrates that the presence of diarrhea during the last 6 months (OR 4.47 95% CI: 1.52, 12.09, *p* = 0.004), a CD4+ T lymphocyte count below 200 count/µL (OR 3.51 95% CI: 1.42, 9.12, *p* = 0.008), as well as not having access to a refrigerator for food storage in the household (OR 3.66 95% CI: 1.52, 9.01, *p* = 0.004) were independently associated with the detection of *C. belli* in stool samples of HIV positive individuals. The abovementioned association with the body mass index, in contrast, was not an independent factor within this subpopulation, as suggested by logistic regression (Figure 2).

### 3.5. Correlations of Cyle Threshold (Ct) Values for C. belli with CD4+ T Cell Count, CD4+/CD8+ T Cell Ratio, and HIV Viral Load

The correlation analysis of *C. belli*-specific cycle threshold (Ct) values in real-time PCR and immune parameters in *C. belli*-positive participants revealed no significant correlations for CD4+ lymphocyte count, the CD4+/CD8+ T cell ratio, or the HIV-1 viral load (ρ = 0.06, *p* = 0.761, ρ = −0.16, *p* = 0.501, and ρ = 0.73, *p* = 0.730, respectively).

## 4. Discussion

The study was conducted to assess associations of epidemiological, clinical as well as immunological features of Ghanian HIV patients and the abundance of co-infections with *C. belli*, a coccidian parasite that is known to cause opportunistic enteric infections in immunosuppressed individuals [1,2,4,5,6,7,8,9,10,11,12,13,14,15,16]. It led to a number of results.

First of all, almost all detected infections with *C. belli* could be associated with HIV infections, with only a single *C. belli* DNA detection in a Ghanaian individuum without known HIV infection. Interestingly, the effects of HIV-associated immunosuppression were so prominent that they superimposed potential relations between demographic factors, sex, and age with *C. belli* infection. The recorded *C. belli* prevalence of 4.2% in the assessed Ghanian HIV-positive population was moderately higher than the globally pooled prevalence of 2.5% [2]. However, this is well in line with previous experience with higher infection rates in resource-limited tropical regions [2,15,29]. Further, the assessment was conducted with a highly sensitive and specific real-time PCR approach [46,51], making it likely that sub-microscopic parasite densities within the investigated stool samples were detected as well.

Focusing on epidemiological features of Ghanaian HIV-positive individuals co-infected with *C. belli*, it seems noteworthy that the lack of a refrigerator was identified as a risk factor for cystoisosporiasis, while no differences were seen for other assessed socioeconomic parameters. Considering the access to a refrigerator as a surrogate parameter for the quality of accessible food safety, the finding is well in line *C. belli*’s fecal-oral transmission route [1] and thus with a higher risk for individuals with poorer access to appropriate food safety measures. Notably, it is an indirect indicator of inappropriate food hygiene because the mere presence or absence of a refrigerator is unlikely to affect fecal-oral transmission as long as additional food-safety-related issues are identical. Interestingly, access to tap water did not relevantly alter the infection risks, which is in line with reports on partly dissatisfying tap water quality, particularly in rural Ghana [53].

Regarding the observed clinical features of cystoisosporiasis in Ghanaian HIV patients, diarrhea, weight loss as well as associated reduced BMI values were recorded. The findings match previous reports [4,5,6,7,8,9,10,11,12,13,14,15] and were insofar not surprising. Considering the robust association of cystoisosporiasis and chronic diarrhea in immunosuppressed individuals [24,25] combined with high detection rates of *C. belli* in Ghanaian HIV patients as recorded in the here-presented study and available anti-parasitic treatment options [36,37,38,39,40,41], a suspicion of cystoisosporiasis should generally be diagnostically addressed in Ghanaian PLWH showing the abovementioned symptoms. In contrast to previous reports [28,29,30], we failed to demonstrate a statistically significant inverse association between antiretroviral therapy and cystoisosporiasis. However, at least a trend pointing in this direction could be observed. Furthermore, CD4+ lymphocyte count was previously described as the more relevant predictive parameter [19].

Focusing on immunological parameters, our study confirmed the established association between cystoisosporiasis and reduced CD4+ T-lymphocyte counts <200 cells/µL with an odds ratio of 3.51 (95%CI: 1.42–9.12) [17,18]. Not surprisingly, these cases of *C. belli* infections and reduced CD4+ T-lymphocyte counts were associated with increased HIV-1 virus loads as well, as poor cellular immune response triggers retroviral replication. Interestingly, the combination of reduced CD4+/CD8+ T-lymphocyte ratios and higher expression of HLA-DR+ CD38+ on CD4+ T-lymphocytes indicated increased immune activation in cystoisosporiasis patients. From animal experiments, it is known that *Cystoispospora* spp. tend to trigger a Th2-associated and regulatory immune response [54]. Notably, other immunological parameters were randomly distributed among the assessed Ghanaian PLWH population.

In the here-presented assessment, *C. belli* parasite density as semi-quantitatively assessed via the Ct-values of positive real-time PCR results in the investigated stool samples neither correlated with HIV load nor with immunological parameters like CD4+ T-lymphocyte count or CD4+/CD8+ T-lymphocyte ratio. This suggests that diagnostic approaches combining high sensitivity and specificity, like real-time PCR [46,51], should be generally preferred for the detection of *C. belli* in the stool of immunocompromised individuals, if available, in order not to miss low parasite loads. If molecular diagnostic options are not available in resource-limited settings, diagnostic attempts based on traditional microscopy [42,43,44] should be considered. Notably, the availability of diagnostic *C. belli* PCR can be challenging even in resource-rich industrialized settings because this parameter is usually lacking in modern commercial multiplex panels [55] in line with its low market share [3].

Regarding the use of cotrimoxazole prophylaxis, it is noteworthy that no association was found with the detection of *C. belli* in stool samples in this cohort of PLWH. As the application of cotrimoxazole prophylaxis in HIV patients is a surrogate parameter for severely disturbed cellular immunity [56], this finding is well in line with previous findings suggesting therapeutic failure in PWLH suffering from cystoisosporiasis in case of persisting low to moderate CD4 T-lymphocyte counts [38]. Similarly, poor effects of cotrimoxazole prophylaxis against cyclosporiasis have recently been shown in a comparable assessment [57]. The interpretation of the results is limited by low sample counts. Hypothetically, immune recovery due to antiretroviral therapy in PWLH might mask the role of cotrimoxazole by independently resolving coccidian co-infections, and further, prophylactic treatment duration and compliance with cotrimoxazole intake might have been heterogenous, resulting in a lack of association in the chosen cross-sectional study design of the present and previous [57] assessments. Nevertheless, the findings suggest that coccidian infections have to be considered in severely immunocompromised PLWH in spite of prophylactic cotrimoxazole prescription.

The study has a number of limitations. First, the retrospective study design and the limited size of the study populations limit the interpretability of assessments with sub-populations due to partly very small sample sizes. Accordingly, weak associations were likely to be overlooked in the here-presented assessments. Second, potential enteric co-infections in the Ghanaian high-prevalence setting [58] might have interfered with the clinical effects of the recorded *C. belli* infections. Third, a more exhaustive list of socioeconomic parameters such as household income, educational attainment, location of residence, and others could have been chosen, which might have enhanced the robustness of our study findings.

## 5. Conclusions

In spite of the abovementioned limitations, the here-presented study confirmed associations of cystoisosporiasis in Ghanaian HIV-patients with reduced CD4+ T-lymphocyte counts and increased HIV viral loads, immune-activation as indicated by reduced CD4+/CD8+ T-lymphocyte ratios as well as higher expression of HLA-DR+ CD38+ on CD4+ T-lymphocytes, a symptom complex comprising diarrhea, weight loss, and reduced BMI, a trend towards not being on antiretroviral medication, and lacking access to food safety procedures as exemplified by storing food in refrigerators. Considering the high rate of *C. belli* detections in Ghanaian HIV patients, chronic diarrhea should result in the diagnostic confirmation or exclusion of cystoisosporiasis, even if cotrimoxazole prophylaxis is applied in severely immunocompromised individuals.

## Figures and Tables

**Table 1:** Demographics, socioeconomic parameters, medical parameters, and clinical symptoms in HIV-infected individuals according to *C. belli* status.

	Variable	HIV Positive *C. belli* Positive, n = 25 (4.2%)	HIV Positive *C. belli* Negative, n = 570 (95.8%)	*p*-Value
Demographics	Age in years ± SD	39.6 ± 8.5	40.6 ± 9.6	0.521
	Female *, n (%)	16 (64.0)	418 (75.2)	0.306
Socioeconomic parameters	Access to tap water, n (%)	10 (40.0)	300 (54.0)	0.245
	Electricity in a household, n (%)	23 (92.0)	519 (93.3)	1.000
	Television in a household, n (%)	19 (76. 0)	459 (82.6)	0.568
	Refrigerator in a household, n (%)	12 (48.0)	411 (73.9)	0.009
	Owning a car, n (%)	1 (4.0)	57 (10.3)	0.497
Medical parameters	Cotrimoxazole prophylaxis, n (%)	6 (26.1)	179 (32.8)	0.651
	Intake of cART, n (%)	6 (24.0)	236 (42.4)	0.095
	Body mass index ± SD	21.3 ± 3.2	23.2 ±4.5	0.038
Clinical symptoms during the last six months	Diarrhea, n (%)	7 (28.0)	33 (5.9)	<0.001
	Abdominal pain, n (%)	2 (8.0)	40 (7.2)	0.700
	Fever, n (%)	4 (16.0)	50 (9.0)	0.277
	Cough, n (%)	4 (16.0)	55 (9.9)	0.307
	Weight loss, n (%)	15 (60.0)	126 (22.7)	<0.001

SD—standard deviation; cART—combined antiretroviral therapy. * Biological sex on the dichotomous level was assessed. This is why only the female sex was exemplarily shown to exclude biological sex-related associations.

**Table 2:** Virological and immunological parameters according to the patients’ *C. belli* status.

Variable	HIV Positive *C. belli* Positive	HIV Positive *C. belli* Negative	*p*-Value
Viral load, log10 copies/ml	5.6 (5.2–6.1)	4.1 (1.6–5.3)	<0.001
CD4+ T cell count/µL	118.0 (77.0–359.0)	348.0 (163.0–570.0)	0.001
CD8+ T cell count/µL	1099.5 (715.0–1657.2)	969.5 (630.2–1374.2)	0.411
CD4+/CD8+ T cell ratio	0.2 (0.1–0.3)	0.4 (0.2–0.7)	0.002
HLA-DR+ CD38+ CD4+ (%)	35.8 (28.9–38.7)	17.5 (10.0–31.4)	0.001
HLA-DR+ CD38+ CD8+ (%)	50.3 (43.3–60.6)	44.2 (28.8–58.4)	0.140
CD57+ CD4+ (%)	19.6 (10.6–25.3)	14.4 (8.6–23.8)	0.234
CD57+ CD8+ (%)	54.8 (43.8–63.3)	47.2 (37.6–57.0)	0.313
PD-1+ CD4+ (%)	50.0 (32.4–62.0)	32.8 (23.2–48.2)	0.112
PD-1+ CD8+ (%)	39.4 (28.1–46.8)	33.5 (22.0–45.5)	0.753
Ki67+ CD4+ (%)	26.5 (25.1–26.6)	13.3 (6.3–30.6)	0.269
Ki67+ CD8+ (%)	12.9 (11.3–14.4)	11.8 (6.5–18.4)	0.787

## Data Availability

All relevant data are provided in the manuscript. Raw data can be made available upon reasonable request.
